# Multiple lines of evidence for identifying potential hazards to fish from contaminants of emerging concern in Great Lakes tributaries

**DOI:** 10.1002/ieam.4561

**Published:** 2021-12-24

**Authors:** Sarah M. Elliott, Daniel J. Gefell, Richard L. Kiesling, Stephanie L. Hummel, Chryssa K. King, Charles H. Christen, Satomi Kohno, Heiko L. Schoenfuss

**Affiliations:** ^1^ US Geological Survey Mounds View Minnesota USA; ^2^ US Fish and Wildlife Service, Cortland New York USA; ^3^ US Fish and Wildlife Service Bloomington Minnesota USA; ^4^ St. Cloud State University, St. Cloud Minnesota USA; ^5^ Loyola University Chicago Illinois USA

**Keywords:** Natural resource management, Organic contaminants, *Pimephales promelas*, Screening values, Surface water

## Abstract

Contaminants of emerging concern (CECs; e.g., pharmaceuticals, flame retardants, pesticides, and industrial chemicals) are omnipresent throughout tributaries to the Great Lakes. Furthermore, CECs are often present at concentrations that are potentially hazardous to aquatic species. Since 2010, we characterized the presence of CECs at 309 sites within 47 Great Lakes tributaries and characterized responses of fathead minnow (*Pimephales promelas*) exposed to river water at a subset of 26 sites within four tributaries. Our work resulted in three independent lines of evidence related to the potential hazards of CEC exposure to fish. First, vulnerability (where vulnerability refers to likelihood) of surface waters to CEC presence was predicted using select watershed characteristics. Second, hazard to fish (where hazard means the potential for adverse biological responses) was predicted using screening values for a subset of CECs. Third, biological responses of fathead minnow exposed to river water in streamside exposures were measured. We assessed the congruence of these three lines of evidence for identifying sites with elevated hazards to CEC exposure. Predicted vulnerability and hazards agreed at 66% of all sites. Where the two indices did not agree, vulnerability often underestimated predicted hazard. When compared with measured biological responses from streamside exposures, predicted hazards agreed for 42% of samples. Furthermore, when predicted hazards for specific effect categories were compared with similar measured biomarkers, 26% and 46% of samples agreed for reproductive and physiological effect categories, respectively. Overall, vulnerability and hazard predictions tended to overestimate the measured biological responses, providing a protective estimate of the potential hazards of CEC exposure to fish. When used together, these three approaches can help resource managers prioritize management activities in minimizing hazards of CEC exposure and can be used by researchers to prioritize studies focused on understanding the hazards of CEC exposure to fish. *Integr Environ Assess Manag* 2022;18:1246–1259. © 2021 The Authors. *Integrated Environmental Assessment and Management* published by Wiley Periodicals LLC on behalf of Society of Environmental Toxicology & Chemistry (SETAC). This article has been contributed to by US Government employees and their work is in the public domain in the USA.

## Key Points

- Contaminants of emerging concern (CECs) are widespread in Great Lakes tributaries surface water, posing a potential hazard to fish health.
- We compare results from three independently developed tools to assess the congruence in findings related to potential hazard of CEC exposure to fish in select Great Lakes tributaries.
- Multiple lines of evidence provide useful information for resource managers to make decisions regarding management activities to reduce exposure of aquatic biota to CECs and for researchers to prioritize studies focused on understanding hazards of CEC exposure to fish.

## INTRODUCTION

Myriad anthropogenic contaminants are being introduced continuously into the environment, including pharmaceuticals, personal care products, pesticides, and industrial chemicals, referred to collectively as contaminants of emerging concern (CECs). Contaminants of emerging concern are omnipresent in aquatic environments (Tang et al., 2020), specifically in US tributaries to the Laurentian Great Lakes (hereafter Great Lakes; Baldwin et al., 2016; Choy et al., 2017; Elliott et al., 2017). However, we currently have a tenuous understanding of the factors that influence the presence of CECs in aquatic environments and how the presence of CECs may affect aquatic biota.

Many variables can influence the presence of CECs in the environment including seasonal, temporal, and land cover and land use factors (Fairbairn et al., 2016; Kiesling et al., 2019). Although some relationships between CEC presence and land cover or other watershed characteristics are well defined (e.g., pharmaceuticals are associated with wastewater effluent), more often the relationship is complex. For example, many CECs are associated with multiple uses resulting in various potential pathways to the environment. This complexity is highlighted in Kiesling et al. (2019), where different combinations of land cover and other watershed characteristics were used to predict the occurrence of different CEC classes in Great Lakes tributaries. Overall, land cover and proximity to point sources were important factors in determining CEC class presence, but the importance of individual factors varied by CEC class. Despite this, land cover and land use within a watershed is not always a good indicator of CEC presence (Battaglin et al., 2018; Cipoletti et al., 2019; Deere et al., 2020). This highlights the need for a multiple lines of evidence approach to assess the potential hazard of CEC exposure to aquatic biota.

The Great Lakes tributaries provide habitat for a diverse array of economically and ecologically important fish species (e.g., lake sturgeon [*Acipenser fulvescens*], walleye [*Sander vitreus*], lake trout [*Salvelinus namaycush*], and perch [*Perca* spp.]). Previous research has documented that CECs—and their transformation products—accumulate in freshwater fish species occupying various niches (e.g., Arnnok et al., 2017; Ramirez et al., 2009). Biological effects associated with exposure to CECs in aquatic biota have been documented in a multitude of laboratory studies (see literature reviews in Brausch & Rand, 2011; Crane et al., 2006; Gefell, Annis, et al., 2019; Gefell, Banda, et al., 2019) and in situ studies (Cipoletti et al., 2019, 2020; Jorgenson et al., 2018; Perkins et al., 2017). Observed effects from in situ studies include elevated stress responses such as increased liver mass and decreased sexual maturity, with more effects observed in fish at sites where complex contaminant mixtures were present. Furthermore, proximity to wastewater treatment plants or combined sewer overflows can be associated with greater potential for CEC hazards to fish (Gefell, Annis, et al., 2019). Despite this knowledge, it is difficult to predict how fish may be affected by CEC exposure at any given site or within a specific watershed. Where environmental data and applicable screening values (SVs) exist, resource managers can use this information to prioritize candidate sites for management actions such as fish passage barrier mitigation or habitat enhancement. Recently developed surface water SVs for 14 CECs (Gefell, Banda, et al., 2019) provide one measure of aquatic life hazard to CEC exposure at specific sites. There are limitations to this approach: (1) The small number of CECs represented by the SVs may underestimate overall hazard to fish in the environment that are exposed to complex mixtures of contaminants; (2) limited resources may inhibit data collection to perform a screening assessment. Despite these limitations, the 14 CECs for which SVs were developed include several of the major CEC classes and can provide relevant and useful information for resource managers or researchers.

Our analysis was driven by the need for a framework to assess CEC presence and potential hazards to fish. Several studies have documented the importance of developing multiple lines of evidence to answer questions related to CEC presence and effects in fish (Jorgenson et al., 2018; Mehinto et al., 2021); yet it remains difficult to make informed decisions about how to prioritize sites for natural resource management and/or monitoring activities. Diamond et al. (2015) developed such a framework for prioritizing sites affected by wastewater effluents. Although the framework was developed for sites affected by specific point sources, the general decision‐making steps can be applied to sites potentially affected by other point or nonpoint sources. Similar to the framework developed by Diamond et al. (2015), we used a combination of predictive tools and on‐site exposure assessments to enhance our understanding of hazards to fish associated with CEC exposure in Great Lakes tributaries.

We used three independent approaches resulting in multiple lines of evidence of potential hazards to fish from CEC exposure in US tributaries to the Great Lakes: predicted vulnerability of surface waters to CEC occurrence, predicted hazards to fish health using SVs, and measured biological responses from fathead minnow (*Pimephales promelas*) on‐site exposure experiments. Our objectives were threefold: (1) assess a range of riverine sites for potential CEC occurrence and effects to fish from CEC exposure using two independently developed tools (vulnerability and hazard), (2) assess the congruence between the vulnerability and hazard predictive tools, and (3) determine the congruence of measured biological responses with vulnerability and hazard predictions at a subset of field sites considered for Objective 1.

## METHODS

### Study area

The Great Lakes, bordering the United States and Canada, include Lakes Superior, Michigan, Huron, Erie, and Ontario. The Great Lakes represent approximately 20% of the world's and approximately 84% of North America's freshwater resources. Land cover varies throughout the Great Lakes basin and includes agriculture, urban, forest, and wetland. The Great Lakes and their tributaries are of great economic, cultural, and ecological significance. The Great Lakes supply more than 48 million people in the US and Canada with drinking water, provide habitat for more than 3000 plant and animal species, and provide economic stability to the region in the form of recreational and industrial opportunities (Great Lakes Commission, 2021).

### Surface water sample collection

**Figure 1:**
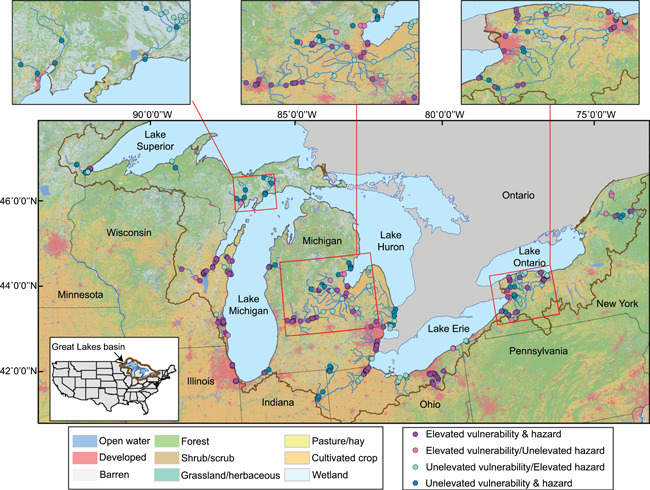
Sites where surface water samples were collected for analysis of contaminants of emerging concern between 2010 and 2019, and vulnerability and hazards to fish were predicted. Land cover adapted from Homer et al. (2015)

Surface water samples were collected between 2010 and 2019. Sample design and CEC determinations varied over time and have been described previously (Elliott et al., 2016, 2017; Lee et al., 2012). Many sites were sampled more than once (typically in spring coinciding with fish spawn and in autumn coinciding with low flow), but most were sampled once. Various subsets of riverine systems were sampled during different years. Briefly, grab surface water samples were collected by dipping baked, amber, glass bottles either directly into the stream or using a weighted bottle sampler. The CECs were characterized at 309 sites representing 47 different riverine systems and a mix of land covers throughout the basin (Figure 1; Table S1).

Samples were analyzed for 110 over‐the‐counter and prescription pharmaceuticals and 64 wastewater indicators (miscellaneous contaminants that may be found in wastewater, such as fragrances or flame retardants) at the US Geological Survey National Water Quality Laboratory in Lakewood, Colorado. These groups of contaminants were chosen because they are frequently detected in the environment and reflect diverse sources of CECs to the environment. Before 2012, 235 whole water samples were analyzed for the presence of pharmaceuticals by extracting samples using methylene chloride in continuous liquid‐liquid extractors and determining pharmaceuticals using capillary‐column gas chromatography/mass spectrometry (Zaugg et al., 2014). Samples collected for pharmaceutical analyses after 2012 (293 samples) were filtered through a 0.7‐µm syringe filter before dispensing into sample bottles. Pharmaceuticals were then determined by direct injection into a high‐performance liquid chromatograph coupled with a triple‐quadrupole tandem mass spectrometer (Furlong et al., 2014). Wastewater indicators were extracted using continuous liquid‐liquid extraction and methylene chloride solvent and determined using capillary‐column gas chromatography/mass spectrometry (Zaugg et al., 2006).

Sites corresponding to on‐site fish exposure experiments (Table S1) were sampled weekly during the exposure, as described in Cipoletti et al. (2019, 2020). Weekly samples were analyzed at SGS AXYS Analytical Laboratories in British Columbia, Canada, for hormones, personal care products, and select pesticides. Although samples from these sites were analyzed for additional CECs (hormones, pesticides), there was substantial overlap with analyses performed on water collected from sites where on‐site fish exposures did not occur (pharmaceuticals, bisphenol A, triclosan). Samples were filtered and cleaned to remove nonspecific matrix interferences using solid‐phase extraction, and contaminants were determined using liquid chromatography/tandem mass spectrometry.

### Vulnerability prediction

To represent the likelihood that CECs may be present at a given site, we calculated a vulnerability score. First, the following watershed characteristics were determined at each site: land cover as a percent of the watershed (e.g., agriculture, forest, developed), number of point sources (hospitals, National Pollutant Discharge Elimination sites, concentrated animal feeding operations, or combined sewer outfalls), distance to the nearest upstream point source, and road density (Table S1; Elliott & Kammel, 2018). For our analysis, the point sources reflect specific potential sources of CECs to the environment, whereas diffuse sources are represented by land cover. Using the attributed characteristics, the probability of CEC presence in water was predicted at each site for eight CEC classes (Table S2; Kiesling et al., 2019) using boosted regression tree models available in Elliott and Kammel (2018): pharmaceuticals, fragrances, industrial chemicals, phosphate‐based flame retardants, organohalides, fecal indicators, phenolics, and plasticizers. An overall measure of vulnerability at each site was calculated by summing the predicted probabilities for all eight CEC classes (Table S2). A score approaching 0 would indicate a relatively low likelihood that any of the CEC classes are predicted to be present, and a score approaching 8 would indicate a relatively high likelihood that all CEC classes are predicted to be present.

### Hazard assessment

The hazard of CEC exposure to fish was determined by assigning scores based on comparisons of aqueous CEC concentrations in water to recently developed SVs for 14 CECs (Table S3; Gefell, Annis, et al., 2019; Gefell, Banda, et al., 2019). These 14 CECs represent those that are frequently detected in the environment and for which adequate toxicological data exist to inform development of SVs. Hazard here is defined as the potential for biological impact. Where sufficient literature ecotoxicity information existed, pairs of SVs (SV_LOW_ and SV_HIGH_) were developed for CEC‐effect category combinations (Gefell, Banda, et al., 2019). In all, 12 effect categories (Table S3) are represented by effect‐specific SVs. For this analysis, 54 SV pairs for 13 CECs were used (Table S3); we excluded DEET (*N*,*N*‐diethyl‐meta‐toluamide) from analysis because it almost always resulted in an elevated hazard that skewed the dataset, making comparison results tenuous. The SV_LOW_ is a concentration in surface water below which hazards to fish are characterized as negligible, whereas the SV_HIGH_ is a concentration in surface water above which hazards to fish are expected. Measured CEC concentrations were assigned an ordinal score representing relative hazard to fish, based on their relationship with the SV_LOW_ and SV_HIGH_ values. Detected concentrations less than SV_LOW_ were assigned a hazard score of 1, and concentrations between SV_LOW_ and SV_HIGH_ were assigned 2; concentrations greater than SV_HIGH_ were assigned 3. All nondetect observations were assigned a hazard score of 1 to indicate negligible hazard. Where detected concentrations were reported by the analytical laboratory as total concentration (unfiltered), the concentration was adjusted to an estimated aqueous concentration before being compared with SVs, using methods detailed in Gefell, Annis, et al. (2019) and Gefell, Banda, et al. (2019). Maximum CEC concentrations determined at each site were compared with 54 SV pairs, resulting in a hazard score database totaling 14 160 scores (Table S4).

### Streamside fathead minnow exposure experiments

Between 2016 and 2019, 21‐day exposure experiments were conducted on selected rivers using a mobile exposure laboratory platform to assess effects of CEC exposure on adult fathead minnow following methods described in Kolok et al. (2012) and Minarik et al. (2014). In all, 26 sites (Table S1) on four tributaries to the Great Lakes were selected for the six streamside exposures: seven sites on the Maumee River (Ohio; 2017 and 2018), six sites on the Milwaukee River (Wisconsin; 2017 and 2018), six sites on the Twin Rivers (Wisconsin; 2019), and seven sites on the Grand River (Michigan; 2019). The on‐site exposure laboratory consisted of an enclosed temperature and photoperiod‐controlled trailer outfitted with aquaria to allow site‐specific exposures of breeding pairs of fathead minnow. All fish exposure protocols were approved by the St. Cloud State University Institutional Animal Care and Use Committee (IACUC permits #8‐82; 8‐107). A daily 50% static renewal procedure was applied using water collected from each field site to minimize sample degradation in the exposure aquaria. The 12 L water collection containers were cleaned using filtered city water (filtered using two carbon filters; Hydronix) between sampling events.

Fathead minnow exposure conditions were modified based on the US Environmental Protection Agency (USEPA; Denny, 1987) and Organisation for Economic Co‐operation and Development (OECD, 2012) guidelines, including consistent temperature (approximately 22 ± 1.3 °C), dissolved oxygen (>5 mg/L), and photoperiod (16 L:8 D). Exposure temperature was altered from USEPA guidelines of 25 °C to 21–23 °C, to approximate ambient temperatures in shallow riverine environments during summer. Mature male and female fathead minnow (6–7 months old) were shipped overnight from Environmental Consulting & Testing and assigned randomly to 3‐L exposure aquaria (fish loading: 3.3–4 g of fish/L). Each aquarium contained one male and one female fish (to allow for the correlation between fish hematological markers and fecundity), one inverted semicircular polyvinyl chloride tile for spawning, and an air stone to maintain dissolved oxygen concentrations above 5 mg/L. Twenty experimental replicates (aquaria) were used at each site. Water quality properties were checked daily using a water‐quality sonde (556 MPS, YSI Incorporated) to record temperature, specific conductivity, total dissolved solids, salinity, dissolved oxygen, pH, and oxidation‐reduction potential. Daily 50% static renewals were performed for all aquaria by siphoning out 1.5 L of water before refilling with appropriate treatment water collected from each field site the day before and allowed to acclimate to ambient temperatures in the exposure trailer overnight. Fish were fed twice daily ad libitum with a pre‐mixed solution of 2:1 frozen brine shrimp:bloodworms (Brine Shrimp Direct) dissolved in filtered city water. Each experiment contained a filtered tap‐water control.

During exposure experiments, aquaria were checked daily for reproduction (fecundity as cumulative mean number of eggs per female per day) and mortality. After 21 days, fish were anesthetized in 0.1% MS‐222 (Argent Chemical Laboratories) and several endpoints were measured to assess fish health: total length, standard length, and wet weight were measured, secondary sexual characteristics (SSC) were scored, and plasma was collected to determine 11‐ketotestosterone, estradiol, glucose, and vitellogenin concentrations (Table S5). Standard length and wet weight were used to calculate the condition factor (CF; [body weight/standard length^3^] × 100). The SSC is the sum of three characteristics that were graded (on a 0–3 scale) blind for the subjective expression of dorsal pad thickness, tubercle presence, and banding coloration; modified from Parrott et al. (2003).

Plasma was collected from male and female fathead minnows by severing the caudal vasculature and collecting blood using heparinized microhematocrit capillary tubes (Fisher Brand). Blood samples were centrifuged at 5000 rpm for 5 min and separated plasma was stored at −80 °C until analyses. Blood glucose was measured using a TRUEbalance blood glucose meter (Moore Medical LLC) within approximately 2 min of fish collection to avoid changes in glucose concentrations associated with stress responses. This analysis required 1 µL of whole blood, making it feasible in the context of maintaining sufficient plasma for protein and hormone analysis. Laboratory analysis of plasma samples for quantification of vitellogenin (Vtg) was conducted using a competitive antibody‐capture enzyme‐linked immunoassay following Parks et al. (1999). Standard preparation and sample analyses followed the previously described methods (Minarik et al., 2014).

### Data preparation

For this study, estimated concentrations (concentrations associated with an “E” remark that typically fall between the detection and reporting levels) were used as reported by the analyzing laboratory. Concentrations qualified as being potentially affected by laboratory contamination were treated as a nondetect. Pharmaceutical results were treated the same, regardless of the analytical method used for determination. Where comparisons could be made, there was generally good agreement in the presence or absence of individual pharmaceuticals between the two analytical methods used throughout the course of sampling (Elliott et al., 2016).

Results from the vulnerability assessment, hazard assessment, and streamside exposure experiments were classified as elevated or unelevated (Table 1). With respect to vulnerability, sites with a vulnerability score greater than 3.8 were classified as having elevated vulnerability to CEC occurrence in surface water, consistent with categories for medium and high vulnerability described in Kiesling et al. (2019), which were based on the quartiles of predicted vulnerability scores during model development. Hazard scores were summarized two ways. First, MaxHaz_Site_ was determined by obtaining the maximum hazard score across 13 CECs and 12 effect categories at each site. The MaxHaz_Site_ was then compared with vulnerability predictions and overall measured biological response from streamside exposures at each site. Second, MaxHaz_Eff_ was determined by obtaining the maximum hazard score for each CEC‐effect category pair at every site. The MaxHaz_Eff_ was compared with specific measured biological endpoints from streamside exposure experiments. The MaxHaz_Site_ and MaxHaz_Eff_ scores of 2 or 3 were classified as elevated hazard to fish, and a hazard score of 1 was considered unelevated.

**Table 1:** Summary of methods used to classify vulnerability scores, hazard scores, and biological responses as elevated

Index	Description	Threshold for elevated classification	Index compared with
Vulnerability	Likelihood of CEC presence at a site	≥3.8	MaxHaz_Site_
MaxHaz_Site_	Maximum hazard score at a site	2, 3	Vulnerability, fish response
MaxHaz_Eff_	Maximum hazard score at a site for each CEC‐effect category pair	2, 3	Endpoint response
Endpoint response	Individual effect endpoint response for each fish	Outside mean ± 1 SD for control fish	MaxHaz_Eff_
Fish response	Overall biological response for each fish	>1 endpoint elevated	MaxHaz_Site_

Abbreviation: SD, standard deviation.

A two‐step approach was used to classify measured biological responses from on‐site exposure experiments as elevated or unelevated (Table 1). First, individual endpoints for each fish were classified as elevated if the value was outside the mean ± 1 standard deviation value for control fish. Second, if more than one individual endpoint was classified as elevated for a fish, that fish was classified as exhibiting an overall elevated response. Using this approach, elevated refers to either a positive or negative numerical change in response, because any deviation from control conditions could be considered potentially detrimental to the exposed organism.

Reproductive response in females was characterized by measurements of plasma Vtg concentration, fecundity, and estradiol concentration; response in males was characterized by plasma Vtg concentration, SSC, and 11‐ketotestosterone concentration. Because several reproductive endpoints were measured, individual endpoints were pooled to indicate an overall reproductive response. Reproductive response was classified in the same way as overall biological response, where fish exhibiting an elevated response in more than one reproductive endpoint (using the mean ± standard deviation method described above) were classified as exhibiting an overall elevated reproductive response.

### Statistical analyses

All statistical analyses were completed in the R environment (v. 4.0.2; R Core Team, 2020). Median concentrations were estimated using the regression on order statistics method in the NADA package (Lopaka, 2020). We used Chi‐square tests (2 × 2 design) to assess associations between vulnerability and hazard scores for all 309 sites that were sampled, and between hazard scores and measured biological responses for all 1382 fish measured at each of the 26 sites where streamside exposures were conducted. For all tests, the null hypothesis was that the variables are independent (e.g., vulnerability and hazard are not associated), and was tested using an alpha level of 0.05. Pearson residuals (observed minus expected frequency divided by the square root of the expected frequency) were calculated to determine which categories contributed most to the significance.

Comparisons of vulnerability and MaxHaz_Site_ indicate whether the two indices align in terms of expected effects on aquatic biota on a site‐by‐site basis. The MaxHaz_Site_ was also compared with the overall measured biological response in fish from streamside exposure experiments. Additionally, MaxHaz_Eff_ for three specific effect categories was compared with relevant measured endpoints for every fish in which it was measured: physiological hazard versus glucose concentrations, growth hazard versus CF, and reproductive hazard versus pooled reproductive endpoints. Comparisons of predicted hazards and measured biological responses indicate whether biological responses in individual fish aligned with predicted hazards at the respective site.

## RESULTS

### Presence of CECs in surface water

**Figure 2:**
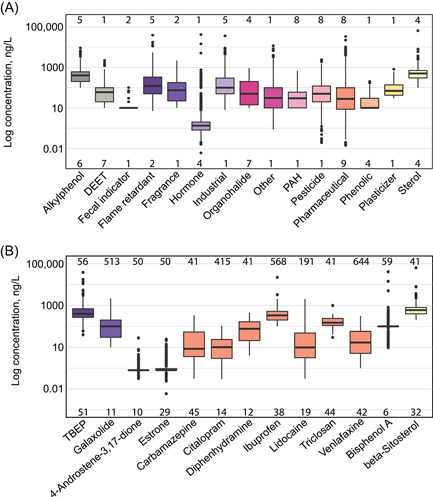
Boxplot summaries of detected concentrations in water by (A) contaminants of emerging concern (CEC) class and (B) select CECs for which screening values exist. Numbers above boxplots indicate how many CECs were detected within that class (A) or sample size (B). Numbers below boxplots indicate the detection frequency in percent. Colors in (B) correspond to CEC class in (A). Median values are represented by the middle line, 25^th^ and 75^th^ percentiles are represented by the bottom and top of the boxes, respectively. Whiskers extend to minimum and maximum values within 1.5 times the interquartile range. Outliers are represented by individual points

A brief description of the CEC data is provided here for context; detailed summaries have been published elsewhere (Choy et al., 2017; Elliott et al., 2017). In all, 27 CECs were detected frequently (≥30% of samples; Table S6). Of the most frequently detected, 13 (48%) were pharmaceuticals (although it is important to note that more pharmaceuticals were analyzed than other CECs). Median concentrations ranged from 3.53 to 600 ng/L, with most <100 ng/L (Figure 2). Several CECs for which SVs exist were among the most frequently detected (e.g., beta‐sitosterol, carbamazepine; Figure 2B). Several of the frequently detected CECs were present in at least half of the sampled rivers, including two industrial chemicals, two pesticides, 12 pharmaceuticals, and two flame retardants. Chemical signatures generally differed, depending on the dominant land cover within the watershed (Elliott et al., 2017). For example, sites located within urban watersheds tended to have more pharmaceuticals and flame retardants than agricultural sites. These results highlight the ubiquitous presence of CECs in US tributaries to the Great Lakes.

### Vulnerability predictions

Predicted vulnerability ranged from 1.8 to 6.7 (possible range of 0–8) across all 309 sites, with 54% of sites classified as having elevated vulnerability to CEC occurrence (Figure 1; Table S2). Vulnerability tended to increase in a downstream manner, reflecting the complex environment in many downstream sites where contaminants can accumulate from various upstream sources. In all, 15 (32%) river systems were predicted to have elevated vulnerability; these river systems have degraded water quality compared with other rivers in the region (e.g., Little Calumet River [Illinois], Cuyahoga River [Ohio], and Fox River [Wisconsin]). Conversely, 13 (28%) river systems were predicted to have unelevated vulnerability to CEC presence. Pharmaceuticals were predicted to occur at 90% of sites, the greatest prevalence among the eight CEC classes for which predictions were made. Conversely, fragrances were predicted to occur the least, at only 30% of sites.

Generally, vulnerability predictions closely matched the observed presence of CEC classes in water samples. The absence or presence of CEC classes was correctly predicted at ≥60% of sites, except for fecal indicators. The presence of fecal indicators was predicted consistently incorrectly with a false positive and false negative rate of 64% and 44%, respectively. In instances when vulnerability predictions did not match observed presence, vulnerability more often overpredicted the presence of CEC classes providing a protective estimate. With the exception of pharmaceuticals, false positive rates ranged from 4% (fragrances) to 62% (fecal indicators) across all CEC classes. In instances where the predicted and observed presence of pharmaceuticals did not match, the presence was always overpredicted. Vulnerability underestimated the presence of fragrances more than any other class, with a false negative rate (fragrances predicted to be absent when they were present in the sample) of 81%.

### Hazard predictions

In all, 227 (73%) of the study sites, representing 42 (89%) of the study river basins, were predicted to have elevated MaxHaz_SITE_ from aqueous exposure to as many as 13 CECs (depending on how many CECs were detected in a sample; Figure 1). Similar to patterns observed with vulnerability predictions, more sites were classified as having elevated hazard in rivers known to have degraded water quality, and unelevated hazard was more likely at upstream sites than at downstream sites in any given river system. Furthermore, more sites located within agricultural or urban watersheds tended to have elevated MaxHaz_SITE_ than sites in wetland or forested watersheds. At least one‐third of all sites within each of 41 (87%) river systems were predicted to have elevated MaxHaz_SITE_. Every site in each of 17 (36%) river systems was predicted to have elevated MaxHaz_SITE_, whereas only five (11%) river systems had no sites with elevated MaxHaz_SITE_. Carbamazepine (pharmaceutical) and galaxolide (hexahydro hexamethyl cyclopentabenzopyran, fragrance) concentrations resulted in the most elevated reproductive and physiological hazards, whereas venlafaxine (pharmaceutical) and beta‐sitosterol (phytosterol) concentrations resulted in the most elevated behavioral hazards.

Only 10 sites did not have elevated MaxHaz_Eff_ in any biological effect category: five on the Twin Rivers (ETR1, ETR3, WTR1, WTR2, WTR3), one on the Maumee in 2017 (MAUWAT1), Beaver Creek in 2017 (a tributary to the Maumee; BCR), and two on the Milwaukee River in 2018 (MEF, MER). Elevated MaxHaz_Eff_ was most often predicted for reproductive effects, followed by developmental and histopathology. This is intuitive because reproductive endpoints integrate adverse effects across multiple biological pathways and often reveal more sensitivity than developmental biomarkers or tissue pathology (Dang et al., 2011). MaxHaz_Eff_ associated with gross pathology and growth biological endpoints varied the least among all sites, with all but one site predicted to be unelevated (KKL on the Milwaukee River system was elevated only during the 2017 exposure). KKL stands out as an outlier in that it has a heavily industrialized watershed with little dilution from contributing sources, compared with other highly urbanized sites. Generally, the Twin Rivers had the fewest sites with elevated MaxHaz_Eff_ for any effect category, and the Grand River had the most.

### Biological responses from streamside exposure experiments

**Figure 3:**
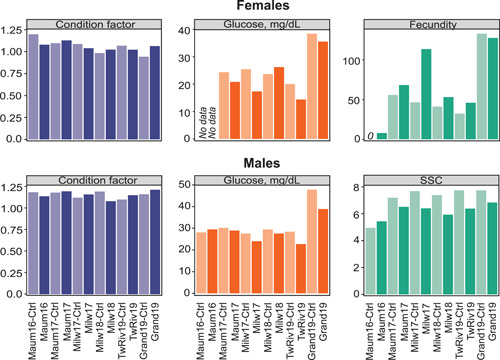
Mean responses of select measured endpoints in female and male fathead minnow after 21‐day on‐site exposure to river water. Lighter shaded bars represent responses in control fish (labeled “XXX‐Ctrl”). Condition factor was calculated as ([body weight/standard length^3^] × 100). Fecundity represents the cumulative mean number of eggs per female per day. Secondary sexual characteristics (SSC) is the sum of three characteristics that were graded (on a 0–3 scale) blind for the subjective expression of dorsal pad thickness, tubercle presence, and banding coloration. Maum16 and Maum17 are Maumee River experiments in 2016 and 2017, respectively. Milw17 and Milw18 are Milwaukee River experiments in 2017 and 2018, respectively. TwRiv19 and Grand19 are the Twin River and Grand River experiments, respectively

Select summary statistics of measured biological responses are provided in Table S6 and Figure 3. Exposure experiments that were conducted in replicate across subsequent years documented both consistency in the association of land use and chemical presence and the variability of chemical concentrations from one year to the next. For most on‐site exposures, fecundity was the most sensitive endpoint measured with increases in females exposed to river water compared with those exposed to control water (Cipoletti et al., 2019; Figure 3). In contrast, physiological endpoints were less often affected. Analysis of all measured biological effects across years and field sites provided little resolution, indicating that the observed effects were either widespread or subtle.

### Comparison of vulnerability and hazard predictions

Comparisons of vulnerability and MaxHaz_Site_ (maximum hazard at each site across all CECs and effect categories) assessed whether predictions of CEC occurrence and predicted hazard to fish are associated. Generally, there was good agreement between vulnerability and hazard predictions (Figure 1), with 66% of sites classified as unelevated or elevated for both indices (*p* < 0.005; Table S7). Sites falling in the unelevated vulnerability and unelevated hazard category contributed most to the significance of the test (Pearson residual = 3.6; Table S7), with more observations than expected. The total number of sites falling into mismatched categories (e.g., elevated hazard and unelevated vulnerability) were observed less often than expected. Of the 104 sites where vulnerability and MaxHaz_Site_ did not agree, 79% were classified as unelevated vulnerability, but elevated MaxHaz_Site_, indicating that vulnerability underestimated the predicted maximum hazard to fish associated with exposure to as many as 13 CECs. Elevated vulnerability at sites predicted to have unelevated hazard occurred at only 7% of sites. No spatial patterns were apparent for sites falling into mismatched categories.

### Comparison of hazard predictions and measured biological responses

**Figure 4:**
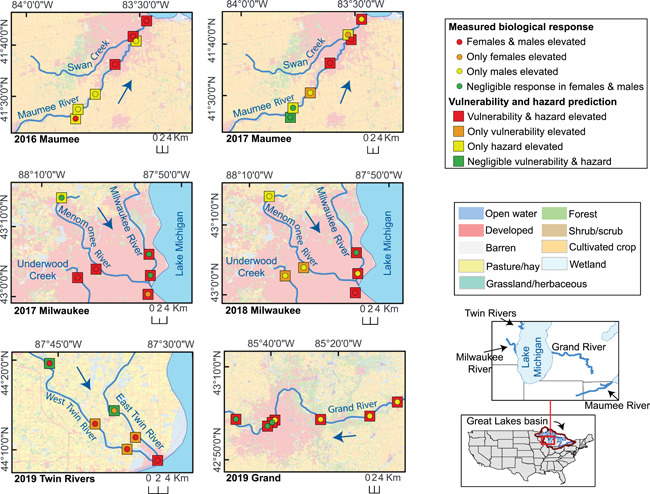
Predicted vulnerability, predicted hazard, and measured biological response at sites where streamside exposure experiments were conducted between 2016 and 2019. Blue arrows indicate the general direction of surface water flow. Land cover adapted from Homer et al. (2015). Km, kilometers

The MaxHaz_Site_ was associated with measured overall biological responses in individual fish (*p* < 0.001; Table S7; Figure 4). Generally, fewer fish were classified in matched categories than mismatched categories; 42% of fish were classified as elevated or unelevated for both hazard and overall biological response. Overall, most observations (42%) fell in the elevated hazard and unelevated biological response category, with the least (9%) falling in the unelevated hazard and unelevated biological response category. Samples classified as unelevated hazard and elevated biological response contributed the most to the significance of the test (Pearson residual = 3.9; Table S7), indicating that more fish were classified as exhibiting elevated responses at sites with unelevated hazard than expected.

MaxHaz_Eff_ was compared with corresponding measured biological responses. Specifically, the physiological MaxHaz_Eff_ was compared with glucose concentrations, growth MaxHaz_Eff_ with CF, and reproductive MaxHaz_Eff_ with pooled reproductive endpoints. The comparison between growth MaxHaz_Eff_ and CF was the only one of the three specific comparisons not to exhibit congruence between the results (*p* > 0.05; Table S7). Approximately 46% and 26% of samples were classified in matched categories for glucose‐physiological MaxHaz_Eff_ and reproductive‐reproductive MaxHaz_Eff_ comparisons, respectively. For both comparisons, observations in which MaxHaz_Eff_ underestimated measured responses contributed the most to the significance of the tests with more observations than expected (Table S7).

## DISCUSSION

Assessing hazards to fish from CEC exposure is inherently difficult given the varied spatial presence of CECs in the environment, the complex mixtures with which CECs occur in the environment, and the inconsistent or muted responses in exposed fish. Such challenges emphasize the importance of using multiple lines of evidence to assess hazards to fish from CEC exposure. We found general congruence among three independent approaches used to estimate the hazard to fish associated with CEC exposure, indicating that an integrated approach can provide valuable information for natural resource managers and other scientists. The information from an integrated approach can provide an indication of sites where CECs are more likely to be present and whether biological effects might be expected, which can be used to prioritize management activities to minimize potential hazards associated with CEC exposure to fish.

Vulnerability and hazard predictions were in agreement for more than half of the sites in our study, indicating that, given a high probability of CEC presence at a site, there is also a high probability of elevated hazards to fish, or, given a low probability of CEC presence, there is also a low probability of elevated hazards to fish. Discrepancies between the vulnerability and hazard indices highlight the intrinsic differences in how the two indices are calculated. First, hazard is based on 13 specific CECs (mostly pharmaceuticals and hormones), whereas vulnerability indicates the potential occurrence of several broad classes of CECs. Furthermore, there is not complete overlap between the CEC classes predicted by the vulnerability tool and the CECs for which SVs exist. For example, in several rivers where vulnerability underestimated predicted elevated hazards, the elevated hazards were mostly attributed to pharmaceuticals (e.g., Vermillion River, Huron River, Johnson Creek). Although the probability of pharmaceutical presence in surface waters in these rivers was high (>0.5), low probabilities of other CEC classes decreased the overall vulnerability index, effectively dampening the potential vulnerability to pharmaceuticals. Second, there were several sites where predicted hazards were elevated because of *beta*‐sitosterol concentrations (e.g., Seney Wildlife Refuge, Irondequoit Bay, Long Pond), but sterols are not accounted for in the vulnerability index.

**Figure 5:**
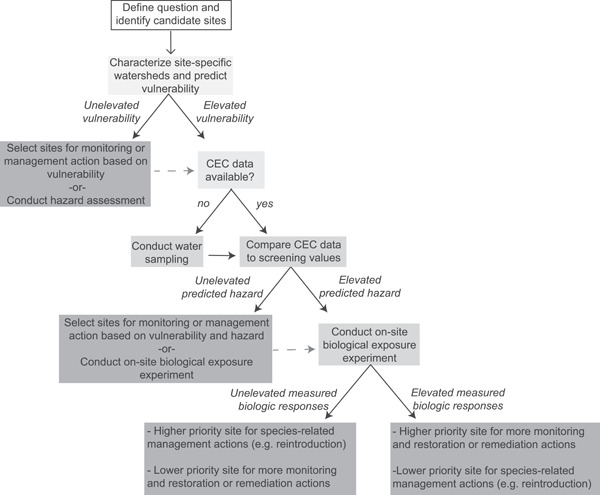
Decision tree showing a framework for use of vulnerability predictions, hazard predictions, and measured biological responses to inform management decisions, monitoring, or research

One limitation of the vulnerability approach is that it only accounts for watershed characteristics and does not account for temporal, hydrologic, or environmental factors. Despite this, the importance of the relationship between land use and CEC occurrence has been documented elsewhere. Watersheds located in areas that are more disturbed tend to have more CECs and higher concentrations in water (Baldwin et al., 2016). Land use and proximity to point sources has been linked to higher CEC concentrations in mussel tissue (Dodder et al., 2014; Maruya et al., 2014) and fish (Deere et al., 2020; Jasinska et al., 2015). Downstream proximity to point sources has also been associated with increased predicted hazard to fish (Gefell, Banda, et al. 2019). Furthermore, although CEC concentrations vary seasonally, their presence in the environment is relatively consistent (Bai et al., 2018) and does not always appear to be related to streamflow (Baldwin et al., 2016). Given the congruence in our findings between vulnerability and hazard, and results from other studies, vulnerability predictions can feasibly be used as a first line of evidence of CEC hazards to fish in tributaries to the Great Lakes. For example, 13 of the 16 tributaries (in common with our study) identified by Corsi et al. (2019) as higher priority for further monitoring based on predicted biological effects were also identified in our analysis as having elevated vulnerability to CEC occurrence. Depending on the question, data availability, and resources, vulnerability predictions can be used directly to inform management actions or to prioritize sites for monitoring and hazard score calculations (Figure 5).

In contrast to the comparison between vulnerability and hazard predictions, we saw less agreement between hazard predictions and measured overall biological responses in on‐site exposed fish. In most cases, hazard overpredicted measured biological responses in individual fish. One hypothesis for the discrepancy is that hazard predictions are generally a more sensitive indicator of potential biological impacts than the on‐site fathead minnow exposures because the SVs were designed to predict hazard to more sensitive fish species than the fathead minnow (Gefell, Banda, et al., 2019). Because biological responses vary among species (Brausch & Rand, 2011; Jorgenson et al., 2018; Thomas et al., 2017), the fathead minnow may be more tolerant of CEC exposure than some of the species tested in assays from which data were used to develop SVs. It is also possible that there was not enough overlap between the endpoints measured from on‐site exposures and endpoints used to develop SVs. Fish from on‐site exposures were assessed for response using seven specific endpoints, whereas SVs were developed using, in some cases, dozens of endpoints. Although measured endpoints were limited compared with those used in SV development, they represent commonly used endpoints that cross multiple levels of biological organization (Hutchinson et al., 2006).

A limitation of the hazard prediction approach is that it does not account for other environmental factors that may affect biological response to CEC exposure. Hazard predictions are based on single‐chemical assays for a limited set of CECs. For example, several CECs that were commonly detected at sites where streamside exposures were conducted (and are also commonly detected in the environment more broadly) were not represented by the SVs, including metolachlor (herbicide), sulfamethoxazole (antibiotic), and tris(2‐chloroethyl) phosphate (flame retardant). Fish in the environment are inherently exposed to multiple stressors, temperature fluctuations, complex chemical mixtures (CECs, nutrients, metals, etc.), and fluctuations in available oxygen. All of these stressors can affect how aquatic organisms respond to their environment, which may differ from how they respond to one of those stressors alone (McBryan et al., 2013; Nilsen et al., 2019).

Hazard predictions are based on measured CEC concentrations in each sample, representing single points in time, which can provide a general indication of potential hazard, but does not account for temporal variability with which CECs may be present in the environment (Bai et al., 2018; Baldwin et al., 2016; Burns et al., 2018). Conversely, because the streamside exposure experiments were conducted for 21 days, they integrated effects from the full range of CECs to which the fish were exposed. Fish could have been exposed to pulses of CECs during which they may have been exposed to a similar concentration as was used for hazard prediction but may have also experienced a period(s) of depuration. Ali et al. (2017) observed a reversal of suppressed biological effects in fathead minnow from a pulse of agricultural runoff following a depuration phase. Similar results have been observed for behavioral alterations in freshwater burbot (*Lota lota*) exposed to high concentrations of oxazepam (anti‐anxiety medication; Sundin et al., 2019). Although these few studies provide some evidence of reversal of compensatory effects in response to CEC exposure, it is important to note they cannot be extrapolated to all CECs or species, so interpretation should proceed with consideration of context. Nonetheless, the studies discussed here do indicate that depuration times and contaminant pulses may be important factors in determining biological responses to CEC exposure and so, in the context of our study, the concentrations used to predict hazard may not sufficiently reflect the exposure scenario in the field.

Despite the differences and limitations discussed here, we saw relatively good agreement among our three approaches, indicating that each has an important role to play in making decisions regarding management actions or research. In most cases, when discrepancies existed among the three approaches, the predictive tools tended to overestimate measured biological responses, indicating they are protective. When considering sensitive species or sites, a protective estimate of potential hazards is ideal. Many fish in the Great Lakes basin are already imperiled and extensive augmentation or reintroduction efforts are underway (e.g., lake sturgeon, lake trout). Because these efforts are costly in both time and funding, it is important to understand the potential hazards associated with CEC exposure at specific sites.

### Limitations and uncertainties

As with any prediction methods, the methods presented in this analysis have inherent limitations and uncertainties. Although many of the uncertainties cannot be quantified, they warrant consideration in the application of these tools and consequent decision‐making related to management activities. One major uncertainty arises from the fact that environmental conditions are not fully accounted for in any of the tools: Vulnerability only incorporates land cover and land use; hazard screening is based on (mostly) laboratory toxicological data, and both hazard screening and on‐site exposure experiments represent one snapshot in time. Additionally, vulnerability was developed and hazard screening was assessed using data collected from grab samples that may miss in‐stream variability of CEC presence. The potential implications of this are that valuable resources may be expended at sites that actually represent a low hazard to aquatic biota, potentially leaving sensitive species at other more affected sites vulnerable to CEC exposure. Because an intensive effort to characterize the presence and effects of CECs is costly and often infeasible, the scientist ultimately needs to make decisions using available data and considering priorities for management actions and allocating resources.

### A framework for prioritizing management actions or monitoring activities

The three approaches presented here (vulnerability, hazard, and on‐site exposure experiments) were initially developed independently of each other. Our assessment indicates congruency in the direction of their findings, and hence their utility to inform management decisions and/or research. These approaches can be used to address questions related to identifying appropriate sites for fish stocking, fish rearing, habitat enhancements, and/or removal of fish passage barriers, to name a few. When used alone, these tools can provide useful information for resource managers and other scientists for answering different scientific questions, but they can also be used in succession as a framework for decision‐making (Figure 5).

When used together, these tools can be applied in the following order: (1) vulnerability prediction, (2) hazard prediction, and (3) on‐site exposure experiments. Results from vulnerability predictions can first be used to determine if and where water sampling may be appropriate to characterize the presence of CECs. A manager or researcher may forgo expensive chemical testing and allocate funding resources based only on vulnerability predictions. If vulnerability predictions are used to prioritize sites for water sampling, the contaminant results can then be compared with SVs, and the results from SV screening (in conjunction with vulnerability predictions) can be used to prioritize sites for project implementation or can be used to identify specific sites for on‐site exposure experiments. This order of approaches to assess hazards to fish from CEC exposure would maximize resource allocation for project implementation and minimize the use of vertebrates in toxicological testing.

## DISCLAIMER

Any use of trade, firm, or product names is for descriptive purposes only and does not imply endorsement by the US government. The findings and conclusions in this article are those of the authors and do not necessarily represent the views of the US Fish and Wildlife Service or the Environmental Protection Agency, but do represent the views of the US Geological Survey.

## Supporting information

This article contains online‐only Supporting Information.

Supporting information regarding sites, chemical data, summary statistics of CECs detected in water, screening value data, vulnerability and hazard predictions, summary statistics of measured biological responses, and frequency tables used in Chi‐square.

## Data Availability

The original data used for analyses presented in this paper are either publicly available or have been published previously: https://pubs.usgs.gov/ds/723, https://doi.org/10.3133/ds910, https://doi.org/10.5066/F7DF6P9D, https://doi.org/10.5066/F7TH8JS6. The specific datasets used for analyses are included in Supporting Information.
